# 

**Published:** 1913-03-22

**Authors:** 


### WORKING-CLASS HOSPITAL ORGANISATIONS.

The Midlands and the North afford far more evidence of strong support for hospitals by the wage-earning classes than is usually to be found in the South. This is well shown by two reports which we select from among many others lately issued. The first is that of the York Workpeople's Hospital Fund, which, in spite of the Insurance Act, collected £1,181 in the year, a minute increase over the figures for 1911. This Fund stimulates the energies of its workers by a system of competitions and prizes. Its economical management is shown by the fact that the total expense of collection was but £21. The other is that of the Leicester Saturday Hospital Society. The report shows that the Society made a record collection for 1912, the grand total being £15,134 13s. Of this amount 70 per cent. was allocated to the Royal Infirmary, and the balance to the convalescent work of the Society. 665 male contributors and 343 female contributors received convalescent benefits in the Society's homes. Referring to the National Insurance Act, the report continues: "The committee do not believe that their splendid organisation is likely to be adversely affected by the new condition of things, which is, after all, only another method of extending the system of insurance which has been popularised in Leicester by the Saturday Hospital Fund." These two organisations are able to show successful results for the past year, but the majority of the reports we have seen lately show diminished collections, ascribed to the effects of National Insurance.

### The Cruelty to Animals Act.

AN ADVISORY COMMITTEE APPOINTED.

In accordance with the recommendation of the Royal Commission on Vivisection, the Home Secretary has appointed an Advisory Committee to assist him in the administration of the Cruelty to Animals Act, 1876. The members of the Committee, who have been selected from names submitted by the Royal Society and the Royal Colleges of Physicians and Surgeons, are:

Sir Anthony Bowlby, C.M.G., F.R.C.S.; Sir John Rose Bradford, K.C.M.G., M.D., D.Sc., F.R.C.P., F.R.S.; Sir Horatio Bryan Donkin, M.D., F.R.C.P.; Mr. George Henry Makins, C.B., F.R.C.S.; the Lord Moulton of Bank; Mr. Seymour J. Sharkey, M.D., F.R.C.P.; and Mr. Charters J. Symonds, M.D., M.S., F.R.C.S.

### THE LONDON MENTAL HOSPITALS COMMITTEE.

It is proposed that the Mental Hospitals Committee of the London County Council during the coming year shall consist of the following members: Mr. T. Chapman, Captain the Hon. A. C. S. Chichester, Mr. W. L. Clague, Colonel R. J. Cooper, Mr. G. Dew, Mr. E. G. Easton, Mr. W. H. Ecroyd, Mr. A. O. Goodrich, Mr. H. J. Greenwood, Mr. G. A. Hardy, Mr. W. Haydon, Mr. T. Hunter, Captain A. C. H. Kennard, Mr. H. Liddiard, Lord Massereene and Ferrard, Mr. E. L. Meinertzhagen, Mr. Henry Mills, Mr. H. E. S. Parsons, Mr. A. C. Rawson, Mr. H. V. Rowe, Lady St. Helier, Mr. R. L. Stuart, Mr. Andrew T. Taylor, Mr. H. R. Taylor, Mr. S. J. Thomas, Sir Edward White, Mr. T. W. Wickham, and Mr. A. W. Yeo.

### Gifts and Bequests.

The Royal Dental Hospital, Leicester Square, W.C., has received a donation of £25 from the Executors of the late Mr. William Kaulla.

Mr. Thomas Parker, of Dartford, subject to his wife's life interest, has left £1,000 to the Earlswood Asylum for Idiots.

Mr. Edward Nightingale, of Rotherham, grocer, subject to one life interest, has bequeathed £1,000 to the Rotherham Hospital and Dispensary.

Miss Maria Frederica Minchin has left £500 to the Rev. Mother of Ascot Priory, Bracknell, Berks, for the convalescent hospital or orphanage attached thereto.

The Niger Company has sent 200 guineas to Mr. Austen Chamberlain for the fund he is raising for the extension of the London School of Tropical Medicine.

Mrs. Maria Bell has left the residue of her estate, which will amount to about £15,000, to the Home for Fatherless Girls, Barrington Road, Brixton, and the Earlswood Asylum for Idiots.

Mr. Nathaniel Louis Cohen, formerly a banker and stockbroker, who left estate valued at £353,152 gross, with net personalty £321,416, has bequeathed £800 to the London Hospital and £1,460 among a number of charities and public objects, Jewish and others.

The Acting Treasurer of St. Bartholomew's Hospital, Mr. G. Acton Davis, acknowledges the receipt of the following: £103 each from the Hospital Saturday Fund and the Peabody Donation Fund (per Lord Dunluce) and £100 each from Sir Everard Hambro, Mr. Arthur S. Bowlby, and Messrs. Blades, East, and Blades.

Mr. Austen Chamberlain acknowledges £250 from Mr. T. Baring Gould, of Meron Court, Guildford; £100 from the Western Telegraph Company, Limited; £100 from Mr. Ernest R. Debenham; and £50 from Messrs. Guthrie and Co. towards the Fund for the Extension and Development of the London School of Tropical Medicine.

### POCKET-BOOK of TEMPERATURE CHARTS.

**Containing seventeen 12-hour and eight 4-hour Charts, perforated so that each Chart may be easily removed after use.**

Bound in paper cover. Suitable size for the pocket. Price **6d.** net; post free, **7d.**

*Of all Booksellers, or of*

THE SCIENTIFIC PRESS, LIMITED, 28 & 29 Southampton Street, Strand, London, W.C.

**If you desire perfect rest and freedom from vibration, get one of**

### LEVESON'S "BERKELEY" CHAIRS

**"YOUR DAUGHTER CAN WHEEL IT."**

Pneumatic Tyres. Cee Springs. Ball Bearings. Soft Upholstery.

**LEVESON & SONS,** SOLE MAKERS,

1, 2, 3, 4 Lawrence Street, High Street Bloomsbury, London, W.C.
26 Knightsbridge, London, S.W.
35 Piccadilly, Manchester.
89 Bold Street, Liverpool.

*Illustrated Catalogue of Invalids' Chairs, Couches, &c., sent post free.*

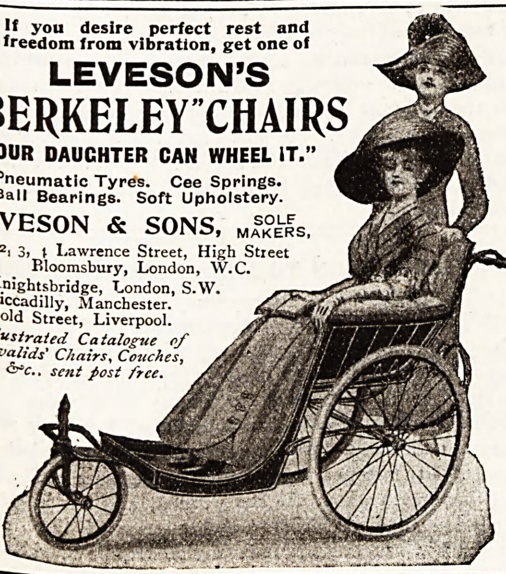

At all Booksellers' and Bookstalls.

### THE BOOK OF DIET

BY

**CHALMERS WATSON, M.D., F.R.C.P.E.**

**2s. Net** **2s. Net**

"The Book of Diet" is a practical guide for the ordinary man as to the forms of diet best suited for various temperaments and occupations, and valuable as a preventive or cure for various diseases.

It is the work of a well-known doctor who is a specialist on the subject

It is not an ordinary medical book, for it is written in popular language and is full of practical illustrations.

"This excellent little volume, written for the laity by a medical authority on diet, contains everyti ing one need know properly to regulate one's diet from infancy to old age, in health and in disease."
*The Daily Mail.*

THOMAS NELSON & SONS,
35 & 35 Paternoster Row, London, E.C., or Parkside, Edinburgh.

### Appointments.

Dr. Thomas Guthrie, F.R.C.S., has been appointed honorary laryngologist to the Liverpool Royal Infirmary.

Mr. W. J. G. Henderson, M.B., Ch.B. (Edin.), has been appointed second house surgeon at Beckett Hospital, Barnsley.

Dr. Daniel Wells Patterson, M.B., B.S., has been elected honorary assistant physician to the Newcastle Royal Victoria Infirmary.

Mr. W. H. Fulford Eales has been elected an honorary surgeon of the Birmingham and Midland Eye Hospital. Mr. Eales' qualifications are: M.A., M.B., B.Ch. (Cantab.), L.R.C.S. (London), M.R.C.S. (England). He is the son of the late Mr. Henry Eales, who was for forty years a member of the honorary staff at the hospital, where he has filled the four resident appointments.

Dr. W. B. Warrington, M.D., F.R.C.P., and Dr. Pantland Hick, M.D., M.B., Ch.B., have been elected honorary physicians to the Liverpool Royal Infirmary. Dr. Warrington graduated at London University in 1894 and obtained the Turner Medical Scholarship at Owens College. He studied at Leipzig and held appointments at the City of London Chest Hospital and at the National Hospital for the Paralysed. Dr. Pantland Hick is also a graduate of London, and his present appointments include honorary physician to the Liverpool Stanley Hospital and honorary assistant physician to the Infirmary for Children.

### Personalia.

Dr. James K. Lennox, of Bright Street, Lochee, is retiring from active practice after fifty-two years. He came from Northumberland in 1862, and his practice has been chiefly among the mill hands of the district. Dr. Lennox is nearly eighty years of age, and will spend his retirement near Macclesfield.

A presentation has been given to Dr. Forsyth, of Braemar, on his departure for York.

Major-General Lee has been re-elected chairman of the board of management of King Edward VII.'s Hospital, Cardiff, for the ensuing year.

Mr. W. Maughan, Mr. Stanley S. Haggie, Mr. John Harker, Mr. A. Selby Wood, Mr. H. Bosh, Mr. Wilson Tyson, the Rev. J. R. Trotter, Canon Gough, and Mr. R. Foreman have been elected to the committee of the Northern Counties Hospital for Consumption and Diseases of the Chest, Newcastle.

Sir William Bull, who has been a member of the committee of the Seaside Convalescent Home, Seaford, Sussex, for twenty-three years, has been re-elected chairman. His re-election was proposed by Mr. George A. D. Goslett.

Mr. E. G. Stone, honorary secretary of the Frimley Cottage Hospital, is leaving the district, though he has been re-elected *pro tem.* to enable the committee to find some one else to take on the work.

The Rev. E. Willis, recently appointed rector of Shadwell, has been elected honorary chaplain to the East London Hospital for Children.

Mr. A. T. Pattison, who, after a year's work, has been re-elected honorary secretary of Macclesfield General Infirmary, received a very warm tribute at the recent annual meeting. Previous to his appointment he was articled to the late Mr. John May, the Father of the Infirmary.

Dr. Roger N. Goodman, who was lately returned unopposed as a member of the Surrey County Council, has been elected a vice-president of the Kingston Victoria Hospital, of which Mr. A. Drinkwater is the hon. secretary.

### Obituary.

Fleet Surgeon R. L. B. Head, late of the Royal Navy, has died at Wimbledon at the age of seventy-nine. After entering the Navy in 1857, having qualified M.R.C.S. in the previous year, as a surgeon he was promoted to the rank of staff surgeon in 1864 for his services at the attack on the batteries in the Straits of Simonoseki, which took place in that year. He was promoted a fleet surgeon in 1877 and retired from the Service in 1878. He had also held the post of surgeon to the Britannia Training Ship for Cadets.

### PRESENTATION TO MISS FISHER AT LEEDS.

"The weekly board of the General Infirmary at Leeds desire to express their great regret at Miss Fisher's retirement from the position of lady superintendent of nurses, which she has occupied for twenty-three years. Miss Fisher's capacity as an administrator, her high sense of duty, and her natural kindness of character have commanded the respect and gained the esteem and affection of those with whom she has worked, and has largely contributed to the efficiency and success of the infirmary itself." Such was the text of the Board's resolution.

